# Emergent Understanding: First Responders’ Feedback to a Dementia Education Training

**DOI:** 10.1093/geroni/igaf122.3577

**Published:** 2025-12-31

**Authors:** Keith Kleszynski, Zachary Chandler, Jack Stanfield, Andrea Golden-Pogue, Shirley James, Thomas Teasdale, Lee Jennings

**Affiliations:** University of Oklahoma Health Campus, Oklahoma City, Oklahoma, United States; The University of Oklahoma, Oklahoma City, Oklahoma, United States; University of Oklahoma College of Medicine, Oklahoma City, Oklahoma, United States; Oklahoma University Health Sciences Center, Oklahoma City, Oklahoma, United States; Hudson College of Public Health, University of Oklahoma Health Sciences, OKLAHOMA CITY, Oklahoma, United States; University of Oklahoma Health Sciences, Oklahoma City, Oklahoma, United States; University of Oklahoma Health Sciences, Oklahoma City, Oklahoma, United States

## Abstract

To address the need for increased first-responder (fire, police, emergency medical services) training in dementia awareness and response, the Oklahoma Dementia Care Network developed a novel first-responder training program, consisting of didactic overview and four case-based, role-playing scenarios featuring common experiences from the field. Training content was developed and refined with input from a first-responder focus group. We describe perceptions from the first 34 participants to complete the training. An inductive, thematic analysis examined open-ended responses; questions explored participant experiences, learning outcomes, and recommendations for future content. Two coders coded responses and collaboratively grouped them into themes. Three themes emerged: importance of a practical training focus, profession-specific applications, and increased understanding and empathy. First-responders emphasized actionable strategies over theoretical knowledge, aligning with the time-sensitive, high-stakes environment they operate in. Participants identified specific communication techniques (i.e., “slowing down,” using body language/positioning, and de-escalation strategies) as elements missing in their current skill sets. After completing training, participants reported increased confidence interacting with PWD (37 percentage point increase, p < 0.01), ability to respond to PWD with understanding (34 percentage point increase, p < 0.01), motivation to get to know PWD better (27 percentage point increase, p = 0.03), 88% learned about new dementia community resources, and 88% rated the training as very good or excellent. A hands-on training to equip first-responders with tools to better navigate emergency response situations with PWD was well-received and filled a learning gap. These findings highlight the practical value of a dementia-focused training developed with first-responder input that resonates with real-world field experiences.

